# Correction: Yau et al. Cardiac and Mental Benefits of Mediterranean-DASH Intervention for Neurodegenerative Delay (MIND) Diet plus Forest Bathing (FB) versus MIND Diet among Older Chinese Adults: A Randomized Controlled Pilot Study. *Int. J. Environ. Res. Public Health* 2022, *19*, 14665

**DOI:** 10.3390/ijerph22040582

**Published:** 2025-04-08

**Authors:** Ka-Yin Yau, Pui-Sze Law, Chung-Ngok Wong

**Affiliations:** 1 School of Nursing, Tung Wah College, Ho Man Tin, Hong Kong; 2 School of Nursing, and Health Studies, Hong Kong Metropolitan University, Ho Man Tin, Hong Kong; 3 Research Office, Tung Wah College, Ho Man Tin, Hong Kong

In the original publication [1], there was a mistake in Table 4 as published. The authors carelessly mentioned the confidence intervals in reverse order in Table 4 and mistakenly reported the square of the effect size (η²) in the original table. In this revision, the correct effect size (η) is reported in the relevant column. The corrected Table 4 appears below. A correction has been made to Section 3.4. Intervention Effect on Other Variables.

The changes in adiposity, cardiovascular risk factors, and psychological indicators in each group and the mean differences between the three groups after the four-week intervention are shown in Table 4. After the four-week intervention, the mean waist-to-hip ratios for the MIND group, MIND-FB group, and control group were 0.885 (95% CI: 0.86–0.91), 0.89 (95% CI: 0.87–0.91), and 0.92 (95% CI: 0.90–0.94), respectively. In a post-hoc comparison, the mean waist-to-hip ratio for the MIND group was 0.003 less than that of the control group (*p* = 0.79). Similarly, the mean waist-to-hip ratio for the MIND-FB group was 0.012 less than that of the control group (*p* = 0.28). The mean BMI demonstrated a significant decrease of 0.24 kg/m^2^ in the MIND-FB group compared to that of the control group. Although all cardiovascular risk factors, trait anxiety levels demonstrated no significant difference between groups, total cholesterol, and lower-density lipoprotein cholesterol (LDL-C) were significantly decreased by 0.61 and 0.34 mmol/L (*p* < 0.01) in the MIND group; 0.86 and 0.59 mmol/L (*p* < 0.01) in the MIND-FB group, and 0.58 and 0.28 mmol/L (*p* < 0.02) in the control group, respectively. Triglycerides (TG) and glucose (Glu) levels were significantly lower at 0.28 mmol/L and 0.68 mmol/L (*p* < 0.05) in the MIND-FB and MIND groups, respectively. The mean difference for state and trait anxiety level, total mood states, tension–anxiety, fatigue, anger, and confusion were significantly lower by 5.28, 4.28, 6.5, 2.2, 1.4, 1.24, and 1.28, respectively (*p* < 0.00) in the MIND-FB group. The mean score of anger–hostility in the MIND_FB group demonstrated a significant 2.32 lower than that of the control group. Also, the mean score of stat anxiety levels in the MIND_FB and MIND groups was significantly lower than that of the control group, by 7.47 and 4 points, respectively. After the four weeks of intervention, the mean score of MIND diet consumption was significantly increased by 3.8 and 3.48 in the MIND and MIND-FB groups, respectively.

The authors state that the scientific conclusions are unaffected. This correction was approved by the Academic Editor. The original publication has also been updated.

## Figures and Tables

**Table 4:** Changes in adiposity, blood pressure, cardiovascular risk factors, mood state, anxiety level, and MIND diet pattern (ITT analysis).

Variable	Mean Changes from Baseline to 4 Weeks After Intervention (95% CI)						*p* Value Across All Groups	Effect Size	MIND vs. Control		MIND-FB vs. Control	
	MIND Group (*n* = 23)	*p* Value #	MIND-FB Group (*n* = 25)	*p* Value #	Control Group (*n* = 24)	*p* Value #			Mean (95% CI) Between Group Difference	*p* Value *	Mean (95% CI) Between Group Difference	*p* Value *
Adiposity												
BMI (kg/m^2^)	−0.04 (−0.14 to 0.05)	0.347	−0.18 (0.39 to 0.01)	0.065	0.05 (−0.15 to 0.25)	0.604	0.013	0.239	−0.09 (−0.33 to −0.15)	0.44	−0.24 (−0.47 to −0.00)	0.047
Waist to hip ratio	−0.02 (−0.04 to 0.01)	0.156	0.00 (−0.01 to 0.01)	0.856	−0.01 (−0.03 to 0.00)	0.070	0.037	0.548	−0.00 (−0.02 to 0.02)	0.79	−0.01 (−0.01 to 0.03)	0.280
Body fat (%)	−0.08 (−0.70 to 0.54)	0.795	−0.69 (−1.50 to 0.12)	0.091	−0.48 (−2.36 to 1.39)	0.599	0.764	0.100	0.41 (−1.30 to 2.11)	0.64	−0.21 (−1.88 to 1.46)	0.800
Blood pressure												
SBP (mmHg)	−1.93 (−6.86 to 2.98)	0.422	−3.18 (−8.70 to 2.34)	0.246	0.67 (−3.11 to 4.44)	0.718	0.490	0.141	−2.61 (−9.24 to 4.03)	0.44	−3.85 (−10.3 to 2.65)	0.240
DBP (mmHg)	−1.26 (−4.51 to 1.99)	0.429	−2.57 (−4.94 to −0.18)	0.036	−0.27 (−2.44 to 1.90)	0.798	0.435	0.152	−0.99 (−4.59 to 0.61)	0.59	−2.29 (−5.82 to 1.24)	0.200
Cardiovascular risk factor												
Total cholesterol (mmol/L)	−0.61 (−0.85 to −0.37)	0.001	−0.86 (−1.34 to −0.37)	0.001	−0.58 (−0.92 to −0.42)	0.002	0.480	0.145	−0.03 (−0.55 to 0.49)	0.91	−0.28 (−0.79 to 0.23)	0.270
LDL-cholesterol (mmol/L)	−0.34 (−0.60 to 0.07)	0.015	−0.59 (−1.01 to −0.17)	0.008	−0.28 (−0.53 to −0.04)	0.026	0.329	0.179	−0.05 (−0.50 to 0.39)	0.81	−0.31 (−0.74 to 0.13)	0.160
HDL-cholesterol (mmol/L)	−0.24 (−0.38 to −0.10)	0.002	−0.14 (−0.24 to −0.05)	0.005	−0.19 (−0.29 to −0.09)	0.001	0.460	0.148	−0.05 (−0.21 to 011)	0.52	0.05 (−0.11 to 0.20)	0.550
Triglyceride (mmol/L)	−0.30 (−0.66 to 0.05)	0.089	−0.28 (−0.54 to −0.02)	0.036	−0.27 (−0.60 to 0.07)	0.116	0.985	0.000	−0.04 (−0.46 to 0.40)	0.86	−0.01 (−0.44 to 0.41)	0.950
Glucose (mmol/L)	−0.68 (−1.34 to −0.03)	0.042	−0.22 (−0.89 to 0.45)	0.507	−0.50 (−1.41 to 0.41)	0.269	0.667	0.110	−0.18 (−1.23 to 0.86)	0.73	0.28 (−0.74 to 1.30)	0.590
Mood states												
POMS total	−1.30 (−6.26 to 3.66)	0.591	−6.56 (−12.1 to 1.03)	0.022	0.13 (−4.20 to 4.45)	0.953	0.120	0.245	−1.43 (−8.03 to 5.44)	0.68	−6.69 (−13.4 to 0.04)	0.051
Tension–anxiety	0.04 (−1.14 to 1.23)	0.940	−2.20 (−3.98 to −0.42)	0.018	−0.38 (−1.61 to 0.86)	0.537	0.059	0.281	0.42 (−1.58 to 2.41)	0.68	−1.83 (−3.70 to 0.13)	0.070
Depression–dejection	0.17 (−0.80 to 1.25)	0.740	−1.36 (−2.96 to 0.24)	0.092	−0.13 (−1.47 to 1.22)	0.849	0.225	0.205	0.30 (−1.59 to 2.19)	0.75	−1.24 (−3.09 to 0.62)	0.190
Fatigue–inertia	−0.91 (−2.22 to 0.40)	0.162	−1.48 (−2.62 to 0.34)	0.013	−0.25 (−1.23 to 0.73)	0.604	0.292	0.187	−0.66 (−2.25 to 0.92)	0.41	−1.23 (−2.78 to 0.32)	0.120
Anger–hostility	−0.26 (−1.38 to 0.85)	0.633	−1.24 (−2.42 to −0.06)	0.039	1.08 (−0.36 to 2.53)	0.135	0.028	0.313	−1.34 (−3.08 to 0.39)	0.13	−2.32 (−4.20 to −0.63)	0.008
Confusion–bewilderment	−0.52 (−1.66 to 0.61)	0.351	−1.28 (−2.08 to −0.48)	0.003	−0.46 (−1.09 to 0.18)	0.149	0.305	0.184	−0.06 (−1.26 to 1.14)	0.92	−0.82 (−2.00 to 0.35)	0.174
Vigor–activity	−0.17 (−1.46 to 1.11)	0.781	0.64 (−0.66 to 1.94)	0.321	0.17 (−0.82 to 1.16)	0.732	0.611	0.118	−0.34 (−2.00 to 1.32)	0.68	−0.47 (−1.15 to 2.10)	0.560
Anxiety level												
STAI-S (score)	−1.78 (−4.29 to 0.73)	0.155	−5.28 (−8.14 to −2.42)	0.001	2.29 (−0.74 to 5.33)	0.132	0.001	0.431	−4.07 (−7.96 to −0.19)	0.04	−7.57 (−11.4 to −3.8)	0.000
STAI-T (score)	−0.78 (−4.31 to 2.74)	0.132	−4.28 (−8.06 to 0.50)	0.028	−0.75 (−2.79 to 1.29)	0.454	0.185	0.219	−0.03 (−4.47 to 4.41)	0.99	−3.53 (−7.88 to 0.82)	0.110

Remarks: # *p* < 0.05 for change during the study determined by paired *t*-test. * *p* value were compared with one-way repeated ANOVA using Tukey HSD; BMI = body mass index; MIND group = The Mediterranean-DASH Intervention for Neurodegenerative Delay diet group; MIND-FB group = The Mediterranean-DASH Intervention for Neurodegenerative Delay (MIND) diet plus forest bathing group; DBP = diastolic blood pressure; HDL = high-density lipoprotein; HSD = honest significant difference; LDL = low-density lipoprotein; ITT = intention-to-treat analysis; SBP = systolic blood pressure; STAI-S = state and trait anxiety inventory-state; STAI-T = state and trait anxiety inventory-trait; POMS = the profile of mood states; Effect size is calculated by r2 coefficient of determination.
