# Supplementary material for: Cardiac and Mental Benefits of Mediterranean-DASH Intervention for Neurodegenerative Delay (MIND) Diet plus Forest Bathing (FB) versus MIND Diet among Older Chinese Adults: A Randomized Controlled Pilot Study
Source: Int J Environ Res Public Health. 2022 Nov 8;19(22):14665. doi: 10.3390/ijerph192214665 (PMC9690744; doi:10.3390/ijerph192214665)
Supplement: Supplementary file 1 [file ijerph-19-14665-s001.zip › ijerph-2020553-supplementary.pdf]

Table S1. Baseline socio-demomgrahic and lifestyle characteristics of study subjects (PP analysis).

		MIND Group (n=22)	MIND-FB Group (n=25)	Control Group (n=23)	*p* Value Across All Groups
Age Group					0.041
	<65 years	9 (39.1)	14 (56.0)	5 (20.8)	
	65 years or above	14 (60.9)	11 (44.0)	19 (79.2)	
Gender					
	Female	18 (81.8)	19 (76)	17 (70.8)	0.631
	Male	4 (18.2)	6 (24)	7 (29.2)	
Educational level					0.092
	Primary or below	8 (36.4)	3 (12)	12 (52.2)	
	Secondary	9 (40.9)	14 (56)	9 (39.1)	
	Associate degree	1 (4.5)	3 (12)	0 (0)	
	Bachelor degree	1 (4.5)	3 (12)	0 (0)	
	Master degree	3 (13.6)	2 (8)	1 (4.3)	
	Doctorate degree/PhD degree	0 (0)	0 (0)	1 (4.3)	
Current employment					0.499
	Employed Full time	3 (13.6)	5 (19.2)	4 (17.4)	
	Employed Partl time	2 (9.1)	2 (7.7)	0 (0)	
	Homemaker	4 (18.2)	6 (23.1)	3 (13)	
	Retired	13 (59.1)	13 (50)	16 (69.6)	
Marital status					0.726
	Married	14 (63.6)	15 (60)	12 (52.2)	
	Single/ Divorced/ Separated/ Widow	8 (36.4)	10 (40)	11 (47.8)	
Frequency for exercise					0.243
	1 to 2 times a week	10 (45.5)	14 (56)	8 (34.8)	
	3 to 4 times a week	5 (22.7)	5 (20)	6 (26.1)	
	5 to 6 times a week	6 (27.3)	3 (12)	9 (39.1)	
	Never	1 (4.5)	3 (12)	0 (0)	
Times for exercise per session					0.174
	> 40 minutes	9 (40.9)	5 (20)	8 (34.8)	
	30-40 minutes	3 (13.6)	9 (36)	3 (13)	
	20-30 minutes	3 (13.6)	3 (12)	8 (34.8)	
	10-20 minutes	3 (13.6)	2 (8)	3 (13)	
	<10 minutes	3 (13.6)	3 (12)	1 (4.3)	
	Never	1 (4.5)	3 (12)	0 (0)	
Smoking Habit					0.343
	Smoke 1-10 cigarettes a day	1 (4.5)	0 (0)	0 (0)	
	Ex-smoker	1 (4.5)	0 (0)	0 (0)	

	Never	20 (90.9)	25 (100)	23 (100)	
Drinking Habit					0.291
	More than fourteen times a week	1 (4.5)	0 (0)	0 (0)	
	Five to seven times a week	0 (0)	1 (4)	0 (0)	
	Once or twice a week	1 (4.5)	3 (12)	0 (0)	
	Ex-drinker	1 (4.5)	0 (0)	0 (0)	
	Never	19 (86.4)	21 (84)	23 (100)	
Relaxation practice					0.259
	Yoga	2 (9.1)	0 (0)	0 (0)	
	Tai chi or qigong	0 (0)	0 (0)	1 (4.3)	
	Stretching exercise	4 (18.2)	5 (20)	5 (21.7)	
	Spend time in nature	3 (13.6)	5 (20)	1 (4.3)	
	Slow deep breathing	3 (13.6)	7 (28)	4 (17.4)	
	Mindfulness meditation	0 (0)	1 (4)	2 (8.7)	
	Listen to music	6 (27.3)	5 (20)	2 (8.7)	
	Never	1 (4.5)	0 (0)	4 (17.4)	
	Others	3 (13.6)	2 (8)	4 (17.4)	
History of Hypertension					0.556
	Hypertension with medication	14 (63.6)	13 (52)	14 (60.9)	
	Hypertension without medication	0 (0)	1 (4)	1 (4.3)	
	Pre-hypertension	8 (36.4)	9 (36)	8 (34.8)	
	Unknown Hypertension	0 (0)	2 (8)	0 (0)	
No. of antihypertensive drugs taken					0.613
	0	8 (36.4)	13 (52)	9 (39.1)	
	1	8 (36.4)	9 (36)	8 (34.8)	
	2	4 (18.2)	1 (4)	5 (21.7)	
	3	1 (4.5)	2 (8)	1 (4.3)	
	4	1 (4.5)	0 (0)	0 (0)	

Data are presented as number (%). p-value was calculated by Pearson Chi-Square test; MIND group = The Mediterranean-DASH Intervention for Neurodegenerative Delay diet group; MIND-FB group = The Mediterranean-DASH Intervention for Neurodegenerative Delay (MIND) diet plus forest bathing group; PP: Per-protocol analysis.

Table S2. Baseline measures of study subjects (PP analysis for Continuous variables).

		MIND Group (n=22)	MIND-FB Group (n=25)	Control Group (n=23)	*p* Value Across All Groups	*p* Value * MIND vs. Control	MIND-FB vs. Control
BMI (kg/m^2)		24.9±3.5	24.9±3.3	25.6±2.9	0.702	0.736	0.755
Waist to hip ratio		0.9±0.04	0.89±0.05	0.93±0.05	0.013	0.080	0.012
Body fat (%)		35.2±6.18	35.2±6.9	37.2±5.02	0.428	0.505	0.484
Lipid Panel							
	Total cholesterol (mmol/l)	5.2±1.2	6.0±1.4	5.4±1.2	0.114	0.856	0.298
	LDL-cholesterol (mmol/l)	2.8±1.1	3.4±1.3	2.8±1.2	0.177	1.000	0.236
	HDL-cholesterol (mmol/l)	1.7±0.5	1.7±0.3	1.8±0.3	0.666	0.933	0.644
	Triglyceride (mmol/l)	1.8±1.1	2.1±1.1	2.0±1.2	0.615	0.864	0.886
Glucose (mmol/l)		6.0±1.3	6.2±1.4	6.4±2.0	0.663	0.641	0.935
BP							
	SBP (mmHg)	138.3±14.3	141±12.0	140.8±16.8	0.792	0.840	0.999
	DBP (mmHg)	85.3±10.7	88.5±9.0	79.2±8.7	0.005	0.092	0.003
Mood state							
	POMS total (score)	38.5±13.3	41.8±11.4	42.1±14.3	0.590	0.622	0.996
	Tension-anxiety	4.0±3.0	6.2±3.9	5.4±3.7	0.140	0.429	0.745
	Depression-dejection	3.2±3.1	4.2±3.5	4.6±3.6	0.392	0.382	0.927
	Fatigue-inertia	6.4±3.7	6.6±3.7	6.7±3.7	0.977	0.975	0.996
	Anger-hostility	4.0±2.5	4.8±2.6	4.0±2.9	0.449	0.544	0.501
	Confusion-bewilderment	11.3±3.0	10.6±1.5	11.2±3.0	0.602	0.981	0.726
	Vigor-activity	9.6±2.9	9.5±3.4	10.1±4.5	0.851	0.897	0.857
Anxiety level							
	STAI-S (score)	40.7±8.5	38.8±8.5	36.3±7.1	0.205	0.180	0.541
	STAI-T (score)	39.9±9.2	42.9±8.9	42.2±9.7	0.534	0.696	0.963
MIND diet pattern							
	Diet scores	5.86±1.9	7.0±2.63	7.3±1.9	0.68	0.068	0.848

Data are presented as mean±SD. *p*-value were compared with one-way MANOVA. MANOVA = multivariate analysis of variance; BMI = body mass index; BP = blood pressure; MIND group = The Mediterranean-DASH Intervention for Neurodegenerative Delay diet group; MIND-FB group = The Mediterranean-DASH Intervention for Neurodegenerative Delay (MIND) diet plus forest bathing group; DBP = diastolic blood pressure; HDL = high-density lipoprotein; LDL = low-density lipoprotein; PP: Per-protocol analysis; SBP = systolic blood pressure; STAI = State and Trait Anxiety Inventory; POMS = The Profile of Mood States.* Tukey honest significant difference (HSD).

Table S3. Changes in Adiposity, Blood pressure, Cardiovascular Risk factors, mood states, anxiety level and MIND diet score (PP analysis).

Variable	Mean Changes from Baseline to 4 Weeks after Intervention (95% CI)						p Value Across All Groups	MIND vs. Control		MIND-FB vs. Control	
	MIND Group (n=22)	*p* Value #	MIND-FB Group (n=25)	*p* Value #	Control Group (n=23)	*p* Value #		Mean (95% CI) Between Group Difference	*p* Value *	Mean (95% CI) Between Group Difference	*p* Value *
Adiposity											
BMI (kg/m2)	-0.04 (-0.05 to 0.14)	0.348	-0.18 (0.01 to 0.38)	0.065	0.05 (-0.25 to 0.15)	0.605	0.643	-0.77 (-3.12 to 1.57)	0.708	-0.79 (-3.07 to 1.48)	0.680
Waist to hip ratio	-0.01 (-0.00 to 0.39)	0.157	0.00 (-0.00 to 0.00)	0.856	-0.01 (-0.00 to 0.02)	0.070	0.018	-0.03 (-0.07 to -0.00)	0.056	-0.03 (-0.07 to -0.00)	0.025
Body fat (%)	-0.08 (-0.56 to 0.72)	0.795	-0.69 (-0.11 to 1.50)	0.091	-0.50 (-1.45 to 2.46)	0.600	0.471	-1.83 (-6.41 to 2.74)	0.605	-2.1 (-6.57 to 2.30)	0.485
Blood pressure											
SBP (mmHg)	-2.02 (-3.12 to 7.18)	0.423	-3.18 (-2.33 to 8.69)	0.246	0.69 (-4.64 to 3.25)	0.718	0.663	-3.7 (-13.8 to 6.2)	0.637	-1.7 (-11.4 to 7.9)	0.905
DBP (mmHg)	-1.31 (-2.08 to 4.72)	0.430	-2.56 (0.18 to 4.93)	0.036	-0.28 (-1.98 to 2.55)	0.799	0.005	5.5 (-0.46 to 11.4)	0.076	8.1 (2.34 to 13.9)	0.004
Cardiovascular risk factor											
Total cholesterol (mmol/l)	-0.60 (0.39 to 0.87)	0.000	-0.85 (0.37 to 1.34)	0.001	-0.60 (0.25 to 0.95)	0.002	0.172	-0.21 (-1.07 to 0.63)	0.811	0.42 (-0.39 to 1.25)	0.433
LDL-cholesterol (mmol/l)	-0.35 (0.07 to 0.62)	0.015	-0.58 (0.17 to 1.00)	0.008	-0.29 (0.03 to 0.54)	0.026	0.277	-0.19 (-0.80 to 0.76)	0.990	0.43 (-.032 to 1.19)	0.365
HDL-cholesterol (mmol/l)	-0.25 (0.10 to 0.39)	0.002	-0.14 (0.04 to 0.23)	0.005	-0.19 (0.09 to 0.30)	0.001	0.735	-0.07 (-0.37 to 0.22)	0.822	-0.09 (-0.38 to 0.20)	0.738
Triglyceride (mmol/l)	-0.31 (-0.05 to 0.68)	0.089	-0.28 (0.02 to 0.53)	0.036	-0.27 (-0.07 to 0.63)	0.116	0.522	-0.20 (-0.98 to 0.57)	0.806	0.16 (-0.59 to 0.91)	0.866

Glucose (mmol/l)	-0.71 (0.03 to 1.39)	0.042	-0.22 (-0.45 to 0.89)	0.507	-0.52 (-0.43 to 1.47)	0.269	0.147	-0.53 (-1.27 to 0.20)	0.199	0.01 (-0.72 to 0.70)	0.999
Mood states											
POMS total	-1.36 (-3.84 to 6.56)	0.592	-6.5 (1.03 to 12.08)	0.022	0.13 (-4.66 to 4.40)	0.953	0.307	-4.38 (-11.7 to 2.97)	0.332	-3.68 (-10.7 to 3.44)	0.435
Tension-anxiety	0.04 (-1.28 to 1.19)	0.940	-2.20 (0.41 to 3.98)	0.018	-0.39 (-0.90 to 1.68)	0.538	0.319	-1.13 (-3.06 to 0.81)	0.348	-0.14 (-2.01 to 1.73)	0.983
Depression-dejection	0.18 (-1.3 to 0.94)	0.740	-1.36 (-0.23 to 2.95)	0.092	-0.13 (-1.27 to 1.53)	0.849	0.269	-1.22 (-3.14 to 0.69)	0.284	-0.99 (-2.84 to 0.87)	0.415
Fatigue-inertia	-0.95 (-0.41 to 2.32)	0.163	-1.48 (0.33 to 2.62)	0.013	-0.26 (-0.76 to 1.28)	0.604	0.719	-0.59 (-2.86 to 1.68)	0.809	-0.71 (-2.90 to 1.49)	0.723
Anger-hostility	-0.27 (-0.89 to 1.44)	0.633	-1.24 (0.06 to 2.41)	0.039	1.13 (-2.64 to 0.38)	0.135	0.608	-0.66 (-2.23 to 0.91)	0.579	-0.31 (-1.83 to 1.22)	0.881
Confusion-bewilderment	-0.54 (-0.64 to 1.73)	0.352	-1.28 (0.48 to 2.07)	0.003	-0.47 (-0.85 to 1.14)	0.149	0.189	-0.11 (-1.50 to 1.72)	0.985	-0.98 (-2.54 to 0.58)	0.295
Vigor-activity	-0.18 (-1.16 to 1.73)	0.781	0.64 (-1.94 to 0.66)	0.321	0.17 (-1.2 to 0.86)	0.732	0.806	-0.67 (-3.12 to 1.77)	0.788	-0.34 (-2.71 to 2.03)	0.938
Anxiety level											
STAI-S (score)	-1.86 (-0.76 to 4.49)	0.155	-5.28 (2.42 to 8.13	0.001	2.29 (-5.56 to 0.77)	0.132	0.270	2.21 (-3.14 to 7.56)	0.586	-1.35 (-6.53 to 3.83)	0.808
STAI-T (score)	-0.81 (-2.88 to 4.51)	0.650	-4.28 (0.49 to 8.06)	0.028	-0.78 (-1.38 to 2.91)	0.454	0.625	-2.28 (-7.90 to 3.34)	0.597	-1.05 (-6.49 to 4.40)	0.890
MIND diet pattern											
Diet scores	3.9 (-5.14 to -2.80)	0.000	3.48 (-4.32 to -2.63)	0.000	0.69 (-1.23 to -0.15)	0.013	0.680	0.15 (-1.21 to 1.53)	0.960	1.04 (-2.37 to 0.28)	0.153

# p < 0.05 for change during the study determined by paried t-test. * p-value were compared with one-way repeated ANOVA using Tukey HSD; BMI = body mass index; MIND group = The Mediterranean-DASH Intervention for Neurodegenerative Delay diet group; MIND-FB group = The Mediterranean-DASH Intervention for Neurodegenerative Delay (MIND) diet plus forest bathing group; DBP = diastolic blood pressure; HDL = high-density lipoprotein; HSD = honest significant difference; LDL = low-density lipoprotein; PP: Per-protocol analysis; SBP = systolic blood pressure; STAI-S = State and Trait Anxiety Inventory-State; STAI-T = State and Trait Anxiety Inventory-Trait; POMS = The Profile of Mood States.
